# Meta-Analysis of Changes in the Number and Proportion of Regulatory T Cells in Patients with Ankylosing Spondylitis

**DOI:** 10.1155/2020/8709804

**Published:** 2020-02-19

**Authors:** Ming Li, Xueping Zhou, Lingling Zhou, Zhichao Yu, Ling Fu, Pei Yang

**Affiliations:** ^1^The First Clinical Medical College, Nanjing University of Chinese Medicine, Nanjing 210023, China; ^2^Jiangsu Provincial Key Laboratory of Pharmacology and Safety Evaluation of Material Medical, School of Pharmacy, Nanjing University of Chinese Medicine, Nanjing 210023, China

## Abstract

Studies on the number and proportion of regulatory T cells (Tregs) in ankylosing spondylitis (AS) patients have been controversial, which has led to a disagreement regarding the role of Tregs in the pathogenesis of AS. To clarify this debate, we conducted a meta-analysis to verify the reported changes in Tregs during AS. We systematically searched the PubMed, Foreign Medical Retrieval System (FMRS), and China National Knowledge Infrastructure (CNKI) web of knowledge databases for eligible articles. A meta-analysis of studies that examined the proportion and number of Tregs among peripheral blood mononuclear cells (PBMCs) and CD4^+^ T cells was performed using Stata software. Further, subgroup analysis was performed based on Treg definition markers and disease activity to identify potential sources of heterogeneity. Forty-seven studies involving a total of 4373 participants were included in the meta-analysis. The Treg/PBMC and Treg/CD4^+^ T cell ratios were significantly lower in AS patients than those in healthy controls (HCs). A subgroup analysis indicated that patients defined by CD4^+^CD25^+/high^, CD4^+^CD25^+^CD127^low/−^, and CD4^+^CD25^+^FOXP3^+^ had much lower Treg/PBMC and Treg/CD4^+^ T cell ratios than HCs. Active AS patients also had a substantially lower proportion of Tregs/PBMCs and Treg/CD4^+^ T cells than HCs. The proportion of Tregs among both PBMCs and CD4^+^ T cells was significantly decreased in AS patients. Treg definition markers and disease activity may influence the proportion of Tregs measured among the PBMC and CD4^+^ T cell populations. Further study of the correlation between AS disease activity and the proportion of Tregs in peripheral blood is needed to determine the physiological role of this association. This study implies that loss of Tregs may play a role in the pathogenesis of AS and helps clarify the contradictory Treg results in AS patients. This trial is registered with PROSPERO (CRD42019147064).

## 1. Introduction

Ankylosing spondylitis (AS) is a chronic, inflammatory, systemic immune disease, which is characterized by inflammation of the spine and the sacroiliac joints. Traditionally, AS has been implicated in association with human leukocyte antigen B27 (HLA-B27) [1]. However, growing evidence suggests that T lymphocyte-associated diseases and CD4^+^ T cells and their subsets are involved in AS development [2]. Regulatory T (Treg) cells are an important component of CD4^+^ T cells that maintain peripheral tolerance and suppress antigen-specific immune responses by secreting transforming growth factor-*β* (TGF-*β*), interleukin-10 (IL-10), and IL-4 to inhibit autoimmunity [3–6].

A growing body of evidence suggests that functional defects in Tregs are present in patients with AS and suggests that this may be due to defects in IL-2, decreased phosphorylation of STAT5, decreased expression of fork box P3 (FOXP3)^+^, and elevated levels of CpG methylation in the CNS2 region of the FOXP3 gene. Treg cells do not control the proliferation of effector CD4^+^ T cells [7, 8], and activation of Tregs has been used to treat AS [9, 10].

Despite this evidence, we have less confidence in the possible beneficial effects of therapeutic Tregs in AS patients. Whether and how Tregs participate in the pathogenesis of AS has not been fully elucidated, and data on Tregs in AS patients has been controversial. At present, studies on Tregs in ankylosing spondylitis focus on the number and function of Tregs in patients with ankylosing spondylitis, but the results were not consistent; a reduction [11–13], elevation [8, 14, 15], and no significant change [16, 17] in the number of Tregs in AS patients have all been reported. The possible reasons for these discrepant results are mainly due to the ambiguity of Treg surface-specific markers or the degree of disease activity at the time of patient detection. In addition, it is necessary to specifically indicate that the number of Tregs is inconsistent. For example, some studies use Treg/PBMC (%), while some use Treg/CD4^+^ T cells (%)—the former focuses on describing changes in the amount of Treg in the peripheral blood, while the latter focuses more on the change in a ratio relative to all T cells.

Given that the quantitative and qualitative changes in Tregs in AS are still unclear, Tregs have been suggested to play an important role in the pathogenesis of AS, and Treg-based immunotherapies show promising potency, here we did a metadata analysis to investigate the proportion of Tregs in PBMC and CD4^+^ T cells in AS patients. Understanding changes in the number of Tregs during AS will help us understand the role of Tregs in the pathogenesis of AS in more detail.

## 2. Materials and Methods

### 2.1. Data Sources and Searches

Three Chinese language databases and five English language databases were widely searched for all relevant results until April 10, 2019. The Chinese language databases were China National Knowledge Infrastructure (CNKI), VIP Database (VIP), and Wanfang Data. The five English language databases were PubMed, ScienceDirect, Foreign Medical Retrieval System (FMRS), EMBASE, and Cochrane Library. We also supplemented the search results using Google Scholar. The literature search strategy used the following terms: English (“regulatory T cells” OR “Treg” OR “CD4^+^CD25^+^ T cell” OR “CD4^+^CD25^high^ T cell” OR “CD25^+^CD127^low^ T cell” OR “CD4^+^CD25^+^FOXP3^+^ T cell”) AND (“Ankylosing spondylitis” OR “AS”) and related Chinese (“qiang zhi xing ji zhu yan” OR “qiang zhi”) AND (“tiao jie xing T xi bao” OR “Treg” OR “CD4^+^CD25^+^ T cell” OR “CD4^+^CD25^high^ T cell” OR “CD25 ^+^CD127^low^ T cell” OR “CD4^+^CD25^+^FOXP3^+^ T cell”).

### 2.2. Study Selection

#### 2.2.1. Inclusion Criteria

The following criteria were used to determine if a search result was included in the analysis: (a) original research (not a commentary), (b) human research, (c) the terms “ankylosing spondylitis” and “regulatory T” (or “Treg”) included in the title or abstract, (d) studies that report the proportion of Tregs in CD4^+^ T cells or peripheral blood of AS patients, and (e) studies that can be found on the Internet; the manuscript is linked from the search site to the full text of the manuscript (PDF or website). At the same time, we validated that all AS patients in the selected study were diagnosed according to the 1984 AS New York revised standard [18].

#### 2.2.2. Exclusion Criteria

The following criteria were used to exclude a search result from the analysis: (a) no raw data on the mean and standard deviation (SD) of the ratio of Tregs in CD4^+^ T cells in PB of AS patients or control subjects and (b) no original information on the number of AS patients or control subjects included in the study. Also, the duplicates between PubMed and Google Scholar search were included only once in the analysis.

### 2.3. Data Extraction

Two independent researchers (Ming Li and Zhichao Yu) extracted data from qualified articles according to the set criteria, cross-checked the data, and, in the case of controversial questions, asked the third commentator to join the discussion to resolve the dispute. The data extraction includes the first author's name, publication year, the number of patients and healthy people, the definition of Treg, Treg frequency, diagnostic criteria, and criteria for determining the active period of AS. The NOS was used to assess the quality of the included studies.

### 2.4. Validity and Quality Assessment

According to the rules and scoring criteria of the Newcastle-Ottawa Quality Assessment Scale (NOS) observational quality assessment tool [19], two authors (ML and ZCY) independently assessed the methodological quality of the included studies. A third author (ZXP) was available to discuss disagreements.

### 2.5. Data Analysis

We used Stata12.0 software (Version 12.0; STATA Corporation, College Station, TX, USA) for the statistical analysis. Heterogeneity was assessed using the *I*^2^-statistic. When the heterogeneity was high (i.e., *p* < 0.05, *I*^2^ > 50%), then a random-effects model was adopted and the source of heterogeneity was analyzed. Publication bias was assessed by the Egger and Begg method. Sensitivity analyses were conducted to test the robustness of the original results.

## 3. Results

### 3.1. Literature Search Results

**Figure 1:**
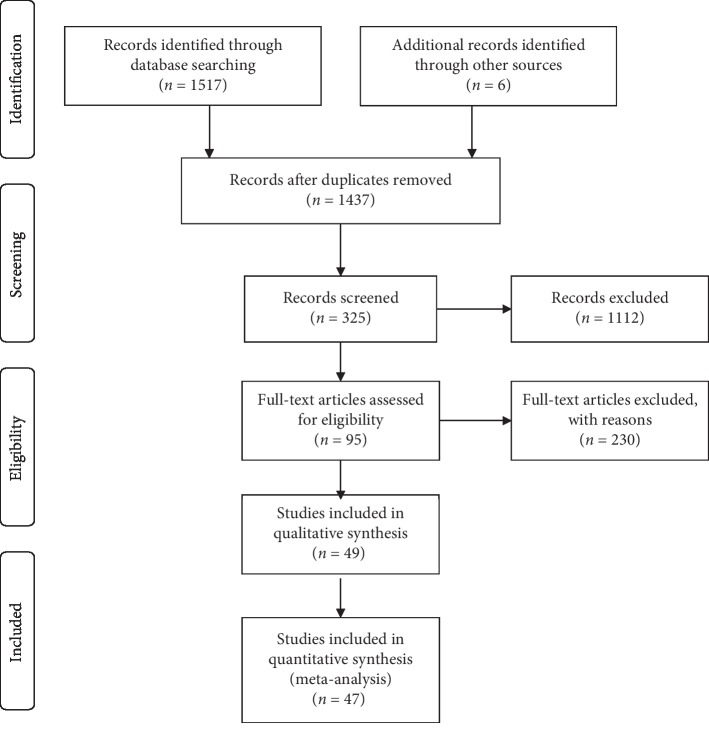
Flow chart of studies included in the meta-analysis.

According to the above search method, 1523 articles were retrieved. A flow chart of the screening process for the articles is shown in Figure 1. First, 86 duplicate articles were excluded. Then, A total of 1112 articles were excluded by screening the titles and abstracts. By reading the full text carefully, articles without original data, controls, or human experiments were excluded, and 47 studies were finally included in the analysis.

### 3.2. Study Characteristics

All of the features included in the study are listed in Tables 1 and 2. This meta-analysis included 2,514 AS patients and 1,859 healthy controls from 47 eligible studies. These studies include one active AS study. Among the 47 included studies, 37 were used to analyze Tregs/PBMCs in AS patients and healthy controls (HCs), 14 were used to analyze Tregs/CD4^+^ T cells in AS patients and HCs, and 4 were used to analyze both Tregs/PBMC and Tregs/CD4^+^ T cell ratios in AS patients and HCs. Criteria for evaluating disease activity and diagnostic criteria were also collected. Based on the quality evaluation criteria of the case-control study based on the NOS, two [31, 49] of the 47 studied scored 4 points, 24 [13–16, 20, 21, 23–26, 30, 32, 36, 37, 39, 42, 44–46, 51, 54, 55, 57, 59] scored 5 points, and 16 [12, 22, 27–29, 33–35, 38, 40, 41, 43, 47, 50, 52, 53, 56, 58, 60] scored 6 points, one [45] of the 7 points. The overall quality of the study is moderate.

### 3.3. Proportion of Tregs in the PB of AS Patients

**Figure 2:**
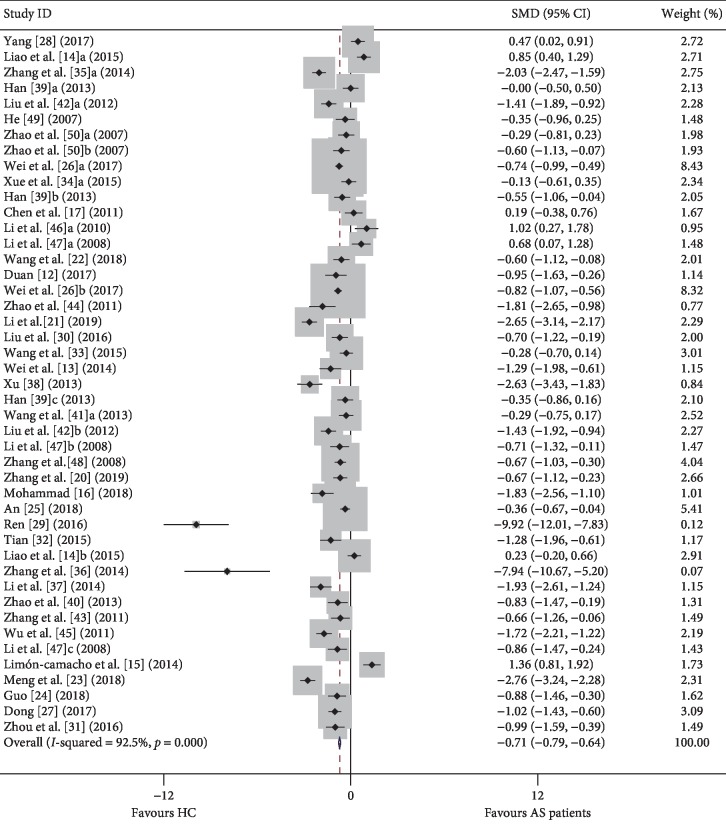
Forest plot of the percentage changes of Tregs in AS patients compared with HCs. SMD: standardized mean difference; CI: confidence interval; AS: ankylosing spondylitis; HC: healthy control; PBMC: peripheral blood mononuclear cell.

First, we compared the proportion of Tregs among PBMCs in the peripheral blood of AS patients and HCs. We initially compared the proportion of Tregs in AS patients and healthy controls regardless of the Treg definition used. In the overall analysis, high heterogeneity (*I*^2^ = 92.50, *p* ≤ 0.001) was observed between the studies, and a random-effects model was used in the meta-analysis. We discovered that the percentage of Tregs in AS was significantly lower than those in controls [−0.071, (−0.79, −0.64), *p* ≤ 0.001] (Figure 2).

We hypothesized that the primary reason for the unexpected results might be that the definitions of Tregs were inconsistent. Thus, we performed a subgroup analysis based on Treg definitions to explore the potential sources of heterogeneity (Table 3). First, we analyzed studies that identified Tregs only as “CD25-positive.” Pooled analysis of all 14 trials revealed a significant decrease in the proportion of Tregs in AS patients compared to controls [−0.369, (−0.493, −0.249), *p* ≤ 0.001] with statistically significant interstudy heterogeneity (*I*^2^ = 91.6%, *p* ≤ 0.001). In detail, we found significant differences in the proportion of Tregs between AS patients and healthy controls when Tregs were defined as “CD4^+^CD25^+^” cells [−0.395, (−0.578, −0.212), *p* ≤ 0.001] and as “CD4^+^CD25^high^” cells [−0.347, (−0.515, −0.178), *p* ≤ 0.001].

Second, the other 14 groups that used “CD127^low/−^” to define Tregs showed that such cell numbers decreased in AS patients [−0.912, (−1.038, −0.786), *p* ≤ 0.001] with statistical heterogeneity (*I*^2^ = 87.3%, *p* ≤ 0.001). More specifically, we found significant differences in the proportion of Tregs between AS patients and healthy controls when Tregs were defined as “CD4^+^CD25^+^CD127^low/−^” cells [−0.855, (−1.065, −0.646), *p* ≤ 0.001] and as “CD4^+^CD25^high^ CD127^low/−^” cells [−0.944, (−1.101, −0.787), *p* ≤ 0.001].

Finally, we analyzed 13 groups in which Tregs were defined as “FOXP3^+^” cells. Pooled analysis of all 13 trials showed that such cell numbers decreased in AS patients [−0.666, (−0.820, −0.512), *p* ≤ 0.001] with statistical heterogeneity (*I*^2^ = 94.5%, *p* ≤ 0.001). More specifically, twelve studies used “CD4^+^CD25^+^FOXP3^+^” to define Tregs, and pooled analysis showed that such cell numbers decreased in AS patients [−0.835, (−0.995, −0.674), *p* ≤ 0.001] with statistical heterogeneity (*I*^2^ = 93.2%, *p* ≤ 0.001). One study used “CD3^+^CD4^+^FOXP3^+^” to define Tregs, so no further analysis was conducted on this study.

To explore the correlation between AS disease activity and the number of Tregs in peripheral blood, we further contrasted the results for active AS patients and HCs (Table 4). We have included a total of 23 studies and found a significant reduction in the proportion of Tregs in patients with active compared to inactive disease [−0.878, (−0.993, −0.762), *p* ≤ 0.001], regardless of the Tregs definitions used. The *I*^2^ values also showed very high heterogeneity (*I*^2^ = 88.0%, *p* ≤ 0.001). Thus, we performed a subgroup analysis based on the Treg definitions to explore the potential sources of heterogeneity in active AS patients. In detail, we found significant differences in the proportion of Tregs between active AS patients and healthy controls when Tregs were defined as “CD25-positive” cells [−0.493, (−0.720, −0.267), *p* ≤ 0.001] with statistical heterogeneity (*I*^2^ = 88.7%, *p* ≤ 0.001). More specifically, Tregs were identified as “CD4^+^CD25^+^” cells [−0.884, (−1.201, −0.567), *p* ≤ 0.001], but no significant difference was apparent when Tregs were defined as “CD4^+^CD25^high^” cells [−0.085, (−0.409, 0.238), *p* ≤ 0.606].

When Tregs were defined as “CD127^low/−^” cells, pooled analysis of all 36 trials revealed a significant difference [−0.889, (−1.086, −0.692), *p* ≤ 0.001] with statistical heterogeneity (*I*^2^ = 83.0%, *p* ≤ 0.001). More specifically, Tregs were identified as “CD4^+^CD25^+^CD127^low/−^” cells [−0.937, (−1.161, −0.714), *p* ≤ 0.001] and as “CD4^+^CD25^high^CD127^low/−^” cells [−0.724, (−1.138, −0.311), *p* ≤ 0.001]. Finally, the other eight groups that used “CD4^+^CD25^+^FOXP3^+^” to define Tregs showed that such cell numbers decreased in active AS patients [−1.180, (−1.397, −0.962), *p* ≤ 0.001] with statistical heterogeneity (*I*^2^ = 91.8%, *p* ≤ 0.001).

We also consider that the evaluation criteria of the AS active period are different, which may be another source of heterogeneity. So, we conducted a subgroup analysis based on the different evaluation criteria of the AS activity period. We found significant differences in the proportion of Tregs between active AS patients and healthy controls when the evaluation criteria of AS active period were based on the Bath Ankylosing Spondylitis Activity Index (BASDAI) [−0.793, (−0.918, −0.667), *p* ≤ 0.001] and the Ankylosing Spondylitis Disease Activity Score (ASDAS) [−1.487, (−1.824, −1.151), *p* ≤ 0.001].

### 3.4. Proportion of Tregs in the CD4^+^ T Cells of AS Patients

**Figure 3:**
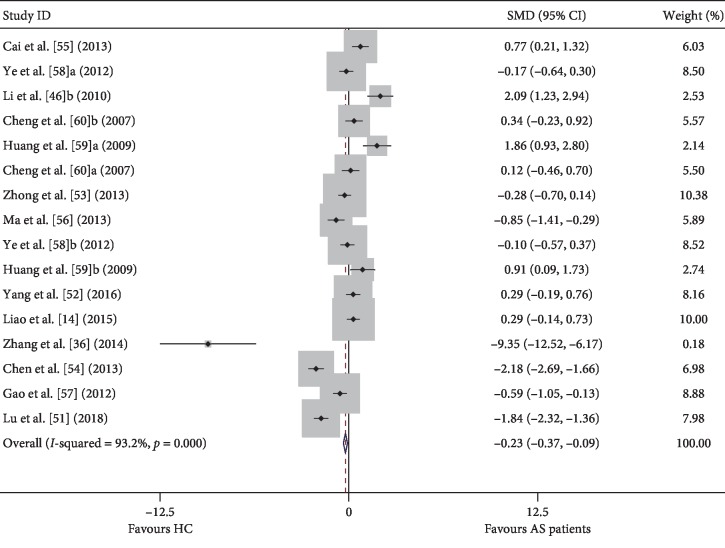
Forest plots generated by meta-analysis for the findings of Tregs/CD4^+^ T cells in AS patients and HCs. SMD: standardized mean difference; CI: confidence interval; AS: ankylosing spondylitis; HC: healthy control; PBMC: peripheral blood mononuclear cell.

In this meta-analysis, among the 47 included studies, 14 were used to analyze Tregs/CD4^+^ T cells in AS patients and HCs. We initially compared the proportion of Tregs in AS patients and healthy controls regardless of the Treg definition used. In the overall analysis, high heterogeneity (*I*^2^ = 93.20, *p* ≤ 0.001) was observed between the studies, and a random-effects model was used in the meta-analysis. We discovered that the percentages of Tregs in AS were significantly lower than those in controls (−0.229, (−0.365, −0.093), *p* ≤ 0.001) (Figure 3). We also performed a subgroup analysis based on the Treg definitions to explore the potential sources of heterogeneity (Table 4).

Tregs were identified as “CD4^+^CD25^+^CD127^low/−^” cells (−0.228, (−0.488, 0.032), *p*=0.006) with statistical heterogeneity (*I*^2^ = 75.5%, *p*=0.163) and Tregs were identified as “CD4^+^CD25^+^FOXP3^+^” cells (−0.494, (−0.727, −0.261), *p* ≤ 0.001) with statistical heterogeneity (*I*^2^ = 95.8%, *p* ≤ 0.001).

To explore the correlation between AS disease activity and the number of Tregs in peripheral blood, we further contrasted results for active AS patients and HCs (Table 4). Similarly, we also explored the correlation between AS disease activity and Treg ratios in CD4^+^ T cells. We found significant differences in the proportion of Tregs between active AS patients and healthy controls when Tregs were defined as “CD4^+^CD25^+^CD127^low/−^” cells (−0.647, (−0.959, −0.336), *p* ≤ 0.001) with statistical heterogeneity (*I*^2^ = 44.9%, *p*=0.163), but no significant difference was apparent when Tregs were defined as “CD4^+^CD25^positive^” cells (0.132, (−0.195, 0.459), *p*=0.428). There was only one study that defined Tregs as “CD4^+^CD25^+^FOXP3^+^”, so no further analysis was conducted for this study. Both ASDAS and BASDAI can be used as a measure of AS disease activity. So, we also conducted a subgroup analysis based on the different evaluation criteria of the AS activity period (Table 4). We found significant differences in the proportion of Tregs between active AS patients and healthy controls when the evaluation criteria of AS active period were based on “BASDAI” (−1.512, (−2.488, −0.535), *p*=0.002) and as “ASDAS” cells (−1.074, (−1.830, −0.318), *p*=0.005) and as “FOXP3^+^” cells (−1.074, (−1.830, −0.318), *p*=0.005).

### 3.5. Publication Bias

**Figure 4:**
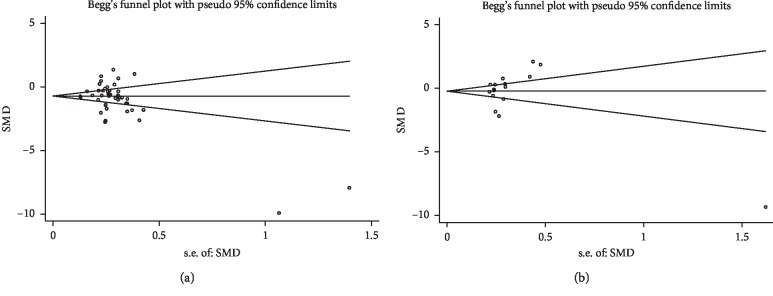
Funnel plot. For interpretation of any publication bias among studies, visual inspection of the generated funnel plot was employed to evaluate symmetry. The funnel plot appears symmetrical.

A meta-analysis of 37 studies on the changes in the number of Tregs in peripheral blood and 14 studies on the changes in the proportion of Tregs in CD4^+^ T cells in patients with AS showed no obvious publication bias by Egger's test ((*t* = −1.91, *p*=0.063) and (*t* = 0.17, *p*=0.870), respectively) (Figure 4).

### 3.6. Sensitivity Analysis

**Figure 5:**
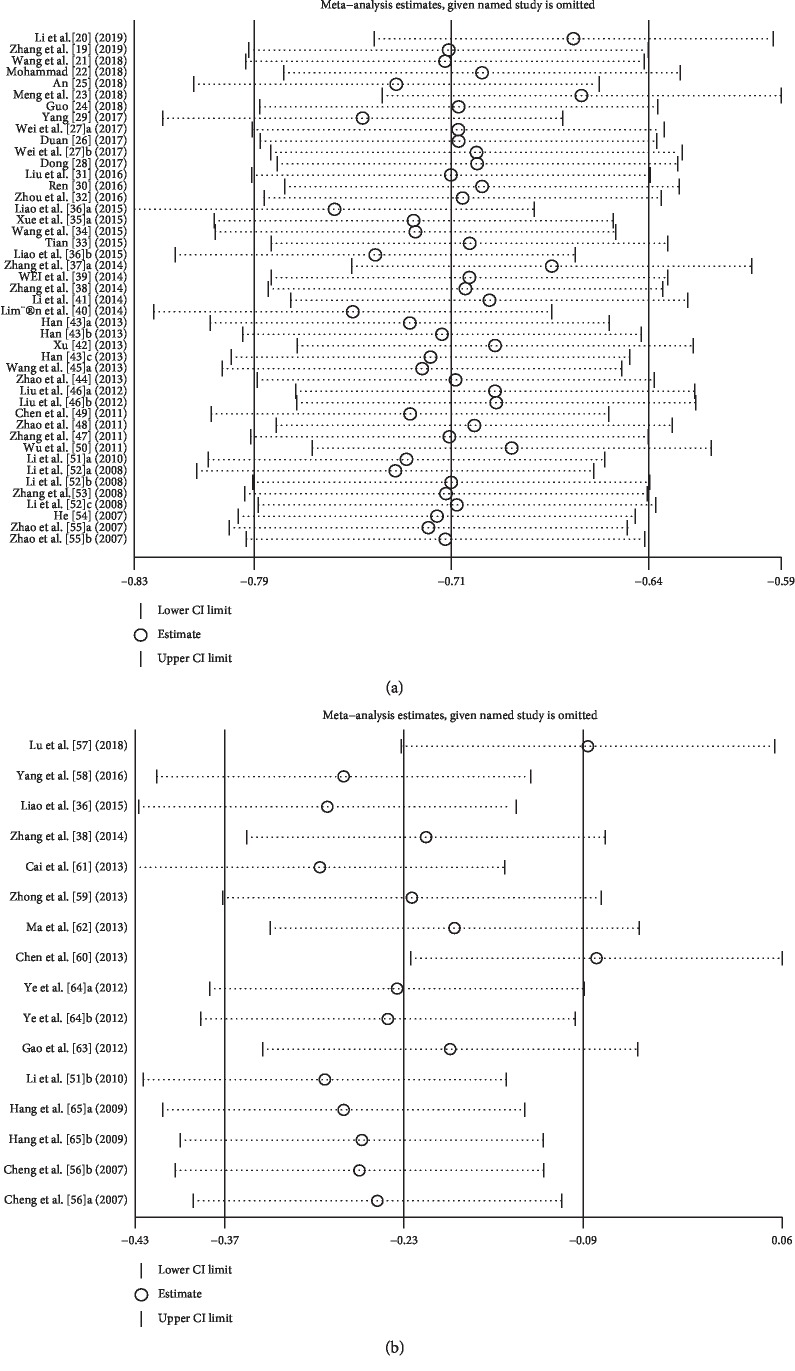
Sensitivity analysis. (a) Sensitivity analysis of Tregs/PBMCs in AS patients and HCs. (b) Sensitivity analysis of Tregs/CD4^+^ T cells in AS patients and HCs.

We conducted a sensitivity analysis to identify possible sources of heterogeneity in studies that determined the proportion of Tregs/PBMC and Tregs/CD4^+^ T cells. Single studies were successively eliminated without any substantial change in the results. This indicated that the results of the meta-analysis were relatively stable (Figure 5).

## 4. Discussion

Regulatory T cells suppress effector cells through a number of secreted and surface-expressed proteins and play an indispensable role in maintaining immune homeostasis and preventing autoimmunity induced by excessive, misleading, or unnecessary immune activation [61, 62]. It is now widely accepted that Treg cells mediate immune tolerance through the immunosuppressive cytokines TGF-β, IL-10, and IL-35. Also, because of the high expression of CD25 in Treg cells, Treg cells are able to clear IL-2, which is a crucial feature [63]. Traditionally, functional defects in Treg cells are thought to play an important role in the pathogenesis of ankylosing spondylitis [64], but the proportion of Tregs in the PBMC or CD4^+^ T cells of AS patients has been controversial, and studies have even suggested that Tregs do not participate in the onset of AS [2].

Our overall meta-analysis concluded that the proportion of Treg in AS patients was significantly lower than that in healthy controls, although significant interstudy heterogeneity was evident. We considered that the primary reasons for such unexpected results were due to various inconsistencies, such as the identification of multiple Treg phenotypes using different markers or inconsistencies in the degree of disease activity.

The most likely reason for the dispute in Treg proportions in AS is that Tregs can be classified into different subgroups. Thus, we subanalyzed the Treg data by the markers used for Treg identification, including CD25, FOXP3, and CD127. There are several definitions of Tregs with different cell surface markers, and this seems to be an important source of heterogeneity.

The understanding of Treg cells is still an evolving process, and CD4^+^CD25^+^ cells were first defined as regulatory T cells in human peripheral blood [65]. Later, FOXP3 proved to be an important marker for regulatory T cells, and it is involved in the establishment and maintenance of Treg cell phenotype [3, 66, 67]. At present, when clinical studies are concerned with the relationship between ankylosing spondylitis and regulatory T cells, it is recognized that regulatory T cells mainly include CD4^+^CD25^+^, CD4^+^CD25^high^FOXP3^+^, and CD4^+^CD25^high^CD127^low^ cells [7, 13].

Tregs were originally described as expressing the peripheral CD4^+^ subpopulation of the IL-2 receptor alpha chain (CD25) [13] and may impair T cell proliferation by depleting local IL-2 concentrations [8, 68]. Further studies indicate that CD25 is expressed not only on Tregs but also on activated cells lacking regulatory functions, although CD4^+^ T cell subsets express the highest levels of CD25 (CD4^+^CD25 ^high^) and exhibit in vitro immunosuppressive properties [15]. Furthermore, it has been reported that CD127 (alpha chain of IL-7 receptor) is upregulated on human T cells after activation and upregulated in Tregs [13], which is inversely correlated with FOXP3 expression levels. Therefore, it has been proposed that staining for CD127 and CD25 can effectively distinguish between Tregs and activated T cells [12]. Classical Treg cells express FOXP3, which is a transcriptional activator of multiple Treg-associated genes [3]. FOXP3, a transcription factor that is expressed at high levels in true Tregs, determines the development and function of Treg cells and is considered to be one of the most specific Treg cell markers [14, 19, 69]. FOXP3 interacts with many other cofactors that are required for the Treg phenotype and function under physiological and pathological conditions [68, 70]. Tregs in peripheral blood from patients with active AS had lower FOXP3 mean fluorescence intensity than those from healthy controls and could not fully suppress naïve T cell proliferation [7].

Although studies have confirmed that ASDAS and BASDAI are highly correlated, they are good measures of AS disease activity [71, 72]. But, BASDAI measures disease activity from the patient's perspective, while ASDAS included not only the patient's opinion but also objective measures, such as C Reactive Protein (CRP), and allows measurement of disease activity at any given time point. So, it does not seem justified to substitute one for the other. The proportion of Tregs in patients with active AS patients was significantly less than that in those with inactive AS, suggesting that Treg cell depletion accelerated disease progression. Our result revealed that the different standard chosen to differentiate active AS from inactive AS could also result in heterogeneity.

Although we also conducted a subgroup analysis of the active AS period according to the different criteria of the activity period, the actual disease evaluation scores are different; some may have very high scores, while some may just meet the activity period judgment criteria. In addition, not only in the study of active AS but also in all studies, the disease state of each patient is unlikely to be at the same level, which may be the main source of heterogeneity.

## 5. Conclusions

In conclusion, our meta-analysis also indicates to a certain extent that the absence of Tregs may play an important role in the pathogenesis of AS. This is also consistent with the performance of Tregs in other immune diseases [64]. This will help us further study the pathogenesis of AS, which will help us to treat AS clinically from the perspective of stimulating Treg value.

## Figures and Tables

**Table 1:** Characteristics of 37 studies used to contrast Tregs/PBMC cells in AS patients and HCs.

Characteristics of 37 studies used to contrast Tregs/PBMCs in AS patients and HCs										
Author (ref.)	Publish year	Country	Tregs' definition	Case numbers (AS/HC)	Tregs/PBMC (%) in AS	Tregs/PBMC (%) in HCs	Age (year) HC/AS	Disease activity score	Diagnosis criteria	NOS score
Zhang et al. [20]	2019	China	CD4^+^CD25^+^FoxP3^+^	60/30	1.65 ± 1.25	2.62 ± 1.76	32 ± 12/29 ± 11	nr	ARA1984	5
Li et al. [21]	2019	China	CD4^+^CD25^high^CD127^low^	64/60	5.11 ± 0.94	9.26 ± 2.03	35.84 ± 6.19/33.26 ± 5.74	nr	ARA1984	5
Wang et al. [22]	2018	China	CD4^+^CD25^high^CD127^low^	21/9	6.84 ± 2.59	8.29 ± 2.25	nr/31.2 ± 4.1	ASDAS ≥ 2.1	ARA1984	6
Fattahi [16]	2018	Iran	CD4^+^CD25^+^FoxP3^+^	35/15	2.70 ± 0.23	3.30 ± 0.47	32.1 ± 8.2/31.4 ± 9.1	BASDAI ≥ 4	ARA1984	5
Meng et al. [23]	2018	China	Treg	34/31	1.23 ± 0.43	3.02 ± 0.81	nr/31.32 ± 2.42	nr	ARA1984	5
Guo [24]	2018	China	Treg	24/27	22.07 ± 12.36	34.05 ± 14.67	30.24 ± 8.24/35.08 ± 9.04	BASDAI ≥ 4	ARA1984	5
An [25]	2018	China	CD4^+^CD25^+^ FoxP3^+^	73/85	30.93 ± 13.59	35.48 ± 12.06	39.63 ± 13.71/38.7 ± 14.23	nr	ARA1984	5
Duan et al. [12]	2017	China	CD4^+^CD25^high^CD127^low^	21/16	2.70 ± 0.80	3.47 ± 0.83	34.6 ± 10.1/37.0 ± 9.8	BASDAI ≥ 4	ARA1984	6
Wei et al. [26]	2017	China	CD4^+^CD25^high^	131/127	4.10 ± 1.12	5.01 ± 1.33	26 ± 9/27 ± 8	nr	ARA1984	5
			CD4^+^CD25^high^CD127^low^	131/127	1.99 ± 1.20	2.99 ± 1.25	26 ± 9/27 ± 8	nr		
Dong [27]	2017	China	Treg	50/50	1.35 ± 0.73	2.64 ± 1.64	43.48 ± 10.48/43.9 ± 10.59	BASDAI ≥ 4	ARA1984	6
Yang [28]	2017	China	CD4^+^CD25^+^	40/40	30.05 ± 5.73	27.4 ± 5.66	33.70 ± 10.06/32.53 ± 9.76	nr	ARA1984	6
Ren et al. [29]	2016	China	CD4^+^CD25^+^FoxP3^+^	48/6	1.23 ± 0.13	2.54 ± 0.15	25.9 ± 4.8/26.2 ± 5.2	nr	ARA1984	6
Lei et al. [30]	2016	China	CD4^+^CD25^+^CD127^low^	60/20	5.68 ± 1.36	6.71 ± 1.75	41.9 ± 11.7/35.0 ± 10.7	nr	ARA1984	5
Zhou [31]	2016	China	Treg	24/24	3.81 ± 1.15	4.98 ± 1.21	24.6 ± 1.2/24.2 ± 1.1	nr	nr	4
Tian [32]	2015	China	CD4^+^CD25^+^FoxP3^+^	30/15	1.25 ± 0.72	2.67 ± 1.64	32 ± 10/35 ± 12	nr	ARA1984	5
Wang et al. [33]	2015	China	CD4^+^CD25^+^CD127^low^	78/30	7.59 ± 1.97	8.16 ± 2.16	25 ± 8/26 ± 7.8	BASDAI ≥ 4	ARA1984	6
Xue et al. [34]	2015	China	CD4^+^CD25^high^	38/30	2.66 ± 1.01	2.80 ± 1.22	30.58 ± 8.39/29.93 ± 9.82	nr	ARA1984	6
				21/30	2.61 ± 1.07	2.80 ± 1.22	nr/29.93 ± 9.82	BASDAI ≥ 4		
Liao et al. [14]	2015	China	CD4^+^CD25^+^	69/30	2.37 ± 0.49	1.97 ± 0.43	nr/39.6 ± 12.7	nr	ARA1984	5
			CD4^+^CD25^+^FoxP3^+^	69/30	1.73 ± 1.08	1.51 ± 0.48	nr/39.6 ± 12.7	nr		
Zhang et al. [35]	2014	China	CD4^+^CD25^+^	60/60	0.98 ± 0.32	2.19 ± 0.78	39.2 ± 3.1/39.0 ± 3.2	nr	ARA1984	6
				18/60	0.75 ± 0.68	2.19 ± 0.78	nr/39.0 ± 3.2	ASDAS ≥ 2.1		
Zhang et al. [36]	2014	China	CD4^+^CD25^+^FoxP3^+^	10/10	1.13 ± 0.17	2.44 ± 0.16	nr	nr	ARA1984	5
Ji et al. [13]	2014	China	CD4^+^CD25^+^CD127^low^	20/20	40.1 ± 17.5	58.6 ± 10.2	nr	nr	ARA1984	5
Limón-camacho et al. [15]	2014	México	CD4^+^FoxP3^+^	39/25	7.30 ± 1.30	5.30 ± 1.70	32 ± 8/32 ± 13	nr	ARA1984	5
Li et al. [37]	2014	China	CD4^+^CD25^+^FoxP3^+^	30/20	3.87 ± 1.11	6.30 ± 1.46	29.2/25.4	nr	ARA1984	5
Xu [38]	2013	China	CD4^+^CD25^+^CD127^low^	24/22	4.23 ± 0.98	6.87 ± 1.03	27.9 ± 8.6/24.3 ± 8.5	ASDAS ≥ 2.1	ASAS	6
Han [39]	2013	China	CD4^+^CD25^high^	30/31	4.69 ± 1.32	5.39 ± 1.24	27.1 ± 6.4/30.6 ± 7.7	nr	ARA1984	5
			CD4^+^CD25^+^	30/31	18.73 ± 5.21	18.74 ± 3.41	27.1 ± 6.4/30.6 ± 7.7	nr		
			CD4^+^CD25^+^CD127^low^	30/31	6.51 ± 1.51	7.08 ± 1.73	27.1 ± 6.4/30.6 ± 7.7	nr		
Zhao and Li [40]	2013	China	CD4^+^CD25^+^FoxP3^+^	21/20	3.90 ± 1.20	4.90 ± 1.20	26 ± 8/24 ± 6	BASDAI ≥ 4	ARA1984	6
Wang et al. [41]	2013	China	CD4^+^CD25^+^CD127^low^	48/36	0.97 ± 0.73	2.12 ± 0.69	23 ± 10/28 ± 9	BASDAI ≥ 4	ARA1984	6
			CD4^+^CD25^+^CD127^low^	37/36	1.89 ± 0.87	2.12 ± 0.69	27 ± 11/28 ± 9	BASDAI ≥ 4		
Jian et al. [42]	2012	China	CD4^+^CD25^+^	60/30	5.90 ± 0.57	6.59 ± 0.26	nr/31.5 ± 9.1	nr	ARA1984	5
			CD4^+^CD25^+^CD127^low^	60/30	1.51 ± 0.26	2.30 ± 0.38	nr/31.5 ± 9.1	nr		
Zhang [43]	2011	China	CD4^+^CD25^+^FoxP3^+^	30/18	1.65 ± 1.25	2.62 ± 1.76	nr/29.64 ± 9.62	BASDAI ≥ 4	ARA1984	6
Zhao et al. [44]	2011	China	CD4^+^CD25^high^CD127^low^	14/18	0.57 ± 0.29	1.65 ± 0.75	28.2 ± 9.4/26.4 ± 6.1	nr	ARA1984	5
Chen et al. [17]	2011	China	CD4^+^CD25^high^	23/25	2.18 ± 0.11	2.16 ± 0.10	33.2 ± 2.25/36.7 ± 3.0	nr	ARA1984	5
Wu et al. [45]	2011	China	CD4^+^CD25^+^FoxP3^+^	51/37	22.23 ± 5.13	32.54 ± 7.05	nr/29.4	BASDAI ≥ 4	ARA1984	7
Li et al. [46]	2010	China	CD4^+^CD25^high^	30/10	5.50 ± 2.73	2.90 ± 1.78	29.2/25.4	nr	ARA1984	5
Li et al. [47]	2008	China	CD4^+^CD25^+^CD127^low/-^	21/24	4.17 ± 1.16	5.01 ± 1.20	29.2 ± 8.0/27.3 ± 12.4	BASDAI ≥ 4	ARA1984	6
			CD4^+^CD25^+^FoxP3^+^	21/24	2.86 ± 1.30	4.00 ± 1.36	29.2 ± 8.0/27.3 ± 12.4	BASDAI ≥ 4		
			CD4^+^CD25^high^	21/24	2.52 ± 1.33	1.79 ± 0.80	29.2 ± 8.0/27.3 ± 12.4	BASDAI ≥ 4		
Zhang et al. [48]	2008	China	CD4^+^CD25^high^CD127^low^	78/50	4.18 ± 1.21	4.99 ± 1.23	25.5 ± 3.8/26.1 ± 6.8	BASDAI ≥ 4	ARA1984	5
He et al. [49]	2007	China	CD4^+^CD25^+^	22/21	1.81 ± 0.97	2.11 ± 0.70	nr	nr	nr	4
Zhao et al. [50]	2007	China	CD4^+^CD25^+^	50/20	8.50 ± 2.70	9.20 ± 1.50	23 ± 9/25 ± 10	BASDAI ≥ 4	ARA1984	6
			CD4^+^CD25^high^	50/20	1.00 ± 0.50	1.30 ± 0.50	23 ± 9/25 ± 10	BASDAI ≥ 4		

AS: ankylosing spondylitis; PBMC: peripheral blood mononuclear cell; HC: healthy control; NOS: Newcastle-Ottawa quality assessment scale; nr: not reported; BASDAI: Bath Ankylosing Spondylitis Activity Disease Activity Index; ASDAS: Ankylosing Spondylitis Disease Activity Score.

**Table 2:** Characteristics of 14 studies used to contrast Tregs/CD4+ T cells in AS patients and HCs.

Characteristics of 14 studies used to contrast Tregs/CD4^+^ T in AS patients and HCs										
Author (ref.)	Publish year	Country	Tregs' definition	Case numbers (AS/HC)	Tregs/PBMC (%) in AS	Tregs/PBMC (%) in HCs	Age (year) HC/AS	Disease activity score	Diagnosis criteria	NOS score
Lu [51]	2018	China	Treg	45/50	1.06 ± 0.28	2.20 ± 0.81	38.1 ± 3.9/38.7 ± 4.2	nr	ARA1984	5
Yang et al. [52]	2016	China	CD4^+^CD25^+^FoxP3^+^	38/31	3.39 ± 0.81	3.15 ± 0.87	29.1 ± 8.1/28.9 ± 10.8	BASDAI ≥ 4	ARA1984	6
Ren et al. [29]	2016	China	CD4^+^CD25^+^FoxP3^+^	48/6	2.82 ± 0.24	5.27 ± 0.28	25.9 ± 4.8/26.2 ± 5.2	nr	ARA1984	5
Liao et al. [14]	2015	China	CD4^+^CD25^+^FoxP3^+^	69/30	4.10 ± 1.47	3.72 ± 0.71	nr/39.6 ± 12.7	nr	ARA1984	5
Naifeng [53]	2015	China	CD4^+^CD25^+^CD127^low^	78/30	7.59 ± 1.97	8.16 ± 2.16	26/27	BASDAI ≥ 4	ARA1984	6
Zhang et al. [36]	2014	China	CD4^+^CD25^+^FoxP3^+^	10/10	2.02 ± 0.21	4.03 ± 0.22	nr	nr	ARA1984	5
Chen et al. [54]	2013	China	CD4^+^CD25^+^FoxP3^+^	61/36	0.94 ± 0.38	2.13 ± 0.75	25 ± 7/25 ± 8.2	nr	ARA1984	5
Cai and Xiao [55]	2013	China	CD4^+^CD25^high^	40/20	11.41 ± 3.69	8.78 ± 2.84	28.4 ± 10.3/29.0 ± 9.4	nr	ARA1984	5
Ma et al. [56]	2013	China	CD4^+^CD25^+^CD127^low^	39/20	6.19 ± 1.51	7.50 ± 1.62	nr/28.3	BASDAI ≥ 4	ARA1984	6
Gao et al. [57]	2012	China	CD4^+^CD25^+^FoxP3^+^	40/37	3.77 ± 1.81	4.69 ± 1.23	26.7 ± 6.9/29.1 ± 8.6	nr	ARA1984	5
Ye et al. [58]	2012	China	CD4^+^CD25^high^	43/30	9.46 ± 4.69	10.89 ± 11.75	32 ± 8/35 ± 10	BASDAI ≥ 4	ARA1984	6
			CD4^+^CD25^+^CD127^low^	43/30	5.89 ± 5.53	6.45 ± 5.72				
Li et al. [46]	2010	China	CD4^+^CD25^high^	30/10	14.6 ± 4.2	6.50 ± 2.60	29.2/25.4	nr	ARA1984	5
Huang et al. [59]	2009	China	CD4^+^CD25^+^	20/9	34.42 ± 12.72	14.2 ± 3.56	nr	nr	ARA1984	5
			CD4^+^CD25^+^CD127^low^	20/9	12.67 ± 5.62	8.31 ± 1.63	nr	nr		
Cheng [60]	2008	China	CD4^+^CD25^+^	25/21	5.62 ± 2.19	5.39 ± 1.70	27 ± 6/28 ± 9	BASDAI ≥ 4	ARA1984	6
			CD4^+^CD25^high^	25/21	1.81 ± 0.83	1.55 ± 0.67	27 ± 6/28 ± 9	BASDAI ≥ 4		

AS: ankylosing spondylitis; PBMC: peripheral blood mononuclear cell; HC: healthy control; NOS: Newcastle-Ottawa quality assessment scale; nr: not reported; BASDAI: Bath Ankylosing Spondylitis Activity Disease Activity Index; ASDAS: Ankylosing Spondylitis Disease Activity Score.

**Table 3:** Subgroup analysis based on Treg definitions.

Tregs definition	Analysis of Tregs/PBMCs						Analysis of Tregs/CD4^+^ T cells					
	Number of studies (*n*)	SMD	95% CI	*P* ^a^	*I* ^2^ (%)	*P* ^b^	Number of studies (*n*)	SMD	95% CI	*P* ^a^	*I* ^2^ (%)	*P* ^b^
CD4^+^CD25^positive^	14	−0.369	−0.493, −0.249	<0.001	91.6	<0.001	6	0.495	0.247, 0.742	<0.001	84.5	<0.001
CD4^+^CD25^+^	7	−0.395	−0.578, −0.212	<0.001	94.8	<0.001	2	0.606	0.113, 1.099	0.016	89.8	0.002
CD4^+^CD25^high^	7	−0.347	−0.515, −0.178	<0.001	84.6	<0.001	4	0.457	0.171, 0.744	0.002	86.5	<0.001
CD4^+^CD25^positive^CD127^low/−^	14	−0.912	−1.038, −0.786	<0.001	87.3	<0.001	—	—	—	—	—	—
CD4^+^CD25^+^CD127^low/−^	4	−0.855	−1.065, −0.646	<0.001	51.2	0.105	4	−0.228	−0.488, 0.032	0.085	75.5	0.006
CD4^+^CD25^high^CD127^low/−^	10	−0.944	−1.101, −0.787	<0.001	90.6	<0.001	—	—	—	—	—	—
CD4^+^CD25^+^FoxP3^+^	12	−0.835	−0.995, −0.674	<0.001	93.2	<0.001	5	−0.494	−0.727, −0.261	<0.001	95.8	<0.001
CD3^+^CD4^+^FoxP3^+^	1	1.363	0.806, −1.919	<0.001	—	—	—	—	—	—	—	—
Treg	4	−1.460	−1.711, −1.208	<0.001	92.2	<0.001	1	−0.229	−0.365, −0.093	<0.001	—	—

**Table 4:** Subgroup analysis based on Treg definitions and the criteria for evaluating disease activity of active AS patients.

Subgroup	Analysis of Tregs/PBMCs						Analysis of Tregs/CD4^+^ T cells					
	Number of studies (*n*)	SMD	95% CI	*P* ^a^	*I* ^2^ (%)	*P* ^b^	Number of studies (n)	SMD	95% CI	*P* ^a^	*I* ^2^ (%)	*P* ^b^
*Tregs definition*												
CD4^+^CD25^positive^	6	−0.493	−0.720, −0.267	<0.001	88.7	<0.001	3	0.132	−0.195, 0.459	0.428	0	0.639
CD4^+^CD25^+^	3	−0.884	−1.201, −0.567	<0.001	91.1	<0.001	1	0.116	−0.465, 0.697	0.696	—	—
CD4^+^CD25^high^	3	−0.085	−0.409, 0.238	0.606	79.7	0.007	2	0.140	−0.256, 0.535	0.489	0.0	0.345
CD4^+^CD25^positive^CD127^low/−^	7	−0.889	−1.086, −0.692	<0.001	83.0	<0.001	—	—	—	—	—	—
CD4^+^CD25^+^CD127^low/−^	5	−0.937	−1.161, −0.714	0.001	88.2	<0.001	3	−0.647	−0.959, −0.336	<0.001	44.9	0.163
CD4^+^CD25^high^CD127^low/−^	2	−0.724	−1.138, −0.311	<0.001	0.0	0.426	—	—	—	—	—	—
CD4^+^CD25^+^FoxP3^+^	8	−1.180	−1.397, −0.962	<0.001	91.8	<0.001	1	0.287	−0.190, 0.763	0.239	—	—
Treg	2	−0.969	−1.307, −0.631	<0.001	0	0.705	—	—	—	—	—	—

*Criteria for evaluating disease activity*												
ASDAS ≥ 2.1	3	−1.487	−1.824, −1.151	<0.001	90.8	<0.001	—	—	—	—	—	—
BASDAI ≥ 4	19	−0.793	−0.918, −0.667	<0.001	87.8	<0.001	—	—	—	—	—	—

*P* ^a^ value refers to association; *P*^b^ value refers to heterogeneity. PBMC: peripheral blood mononuclear cell; SMD: standardized mean difference; CI: confidence interval; *I*^2^ (%): *I*-squared index.
